# High Prevalence of Virulence and *bla_OXA_* Genes Encoding Carbapenemases Among *Acinetobacter baumannii* Isolates from Hospitalised Patients in Three Regions of Poland

**DOI:** 10.3390/pathogens14080731

**Published:** 2025-07-24

**Authors:** Magdalena Szemraj, Małgorzata Piechota, Kamila Olszowiec, Jolanta Wicha, Agata Pruss, Monika Sienkiewicz, Małgorzata Witeska, Piotr Szweda, Barbara Kot

**Affiliations:** 1 Department of Pharmaceutical Microbiology and Microbiological Diagnostics, Medical University of Lodz, 90-151 Łódź, Poland; magdalena.szemraj@umed.lodz.pl (M.S.); kamila.olszowiec@umed.lodz.pl (K.O.); monika.sienkiewicz@umed.lodz.pl (M.S.); 2 Institute of Biological Sciences, Faculty of Exact and Natural Sciences, University of Siedlce, 14 Bolesława Prusa Str., 08-110 Siedlce, Poland; malgorzata.piechota@uws.edu.pl; 3 Medical Microbiological Laboratory, Our Lady of Perpetual Help Hospital, 1/3 Gdyńska Str., 05-200 Wołomin, Poland; jolawich@wp.pl; 4 Department of Laboratory Medicine, Pomeranian Medical University, 70-111 Szczecin, Poland; agata.pruss@pum.edu.pl; 5 Department of Animal Environment Biology, Institute of Animal Science, Warsaw University of Life Sciences—SGGW, Ciszewskiego 8, 02-786 Warsaw, Poland; malgorzata_witeska@sggw.edu.pl; 6 Department of Pharmaceutical Technology and Biochemistry, Faculty of Chemistry, Gdańsk University of Technology, 11/12 G. Narutowicza Str., 80-233 Gdańsk, Poland; piotr.szweda@pg.edu.pl

**Keywords:** antibiotic resistance, genes encoding carbapenemases, virulence genes, biofilm formation

## Abstract

Infections caused by *Acinetobacter baumannii* are increasing worldwide. We evaluated the antibiotic resistance profile, biofilm production, and the frequency of 12 genes encoding carbapenemases and 13 virulence factors in 90 isolates from patients of three hospitals in various regions of Poland. Antibiotic resistance survey was performed using the disc-diffusion method, genes encoding resistance to carbapenems and virulence factors were detected with PCR, and biofilm formation was tested using microtiter plates. A total of 52.2% of isolates were resistant to all tested antibiotic groups (penicillins with β-lactamase inhibitors, cephalosporins, carbapenems, aminoglycosides, fluoroquinolones, and trimethoprim plus sulfamethoxazole). Among the genes encoding carbapenem resistance, the *bla*_OXA-23_ (68.9%), *bla*_OXA-40_ (83.3%), and ISAba-*bla*_OXA-51_ (18.9%) were detected. The *ompA*, *ata,* and *recA* genes responsible for biofilm formation, adhesion, and stress response, respectively, occurred in all isolates. Genes responsible for the production of other adhesins (*bap*—94.4%, *espA*—4.4%, *chop*—37.7%), biofilm formation (*pbpG*—90.0%), production of siderophore (*basD*—97.7%), toxins (*lipA*—92.2%, *cpaA*—1.1%), glycoconjugates (*bfmR*—84.4%), and inducing host cell death (*fhaB*—71.1%, *abeD*—93.3%) were also found. A total of 68.8% of isolates produced biofilm. The isolates from Masovia had more virulence genes than isolates from the other regions; moreover, all isolates from Masovia and West Pomerania were multidrug-resistant (MDR), including resistance to carbapenems.

## 1. Introduction

Increased infections caused by the Gram-negative bacilli *A. baumannii* are currently observed worldwide. This is facilitated by the widespread occurrence of these bacteria in the environment. They may also be present on human skin and colonize livestock [1,2,3]. According to the European Antimicrobial Resistance Surveillance Network, estimated EU incidence of invasive isolates *Acinetobacter* spp. in 2023 was 4.6 per 100,000 population. The percentage of invasive isolates resistant to at least one of the antimicrobial groups (fluoroquinolones, aminoglycosides, or carbapenems) ranged between 0.0% and 96.6% depending on the country. The highest resistance to fluoroquinolones occurred (42.4%), followed by carbapenems (40.1%) and aminoglycosides (36.7%). The percentage of strains resistant to carbapenems in the countries of Southern and Eastern Europe exceeded 50%, whereas in the countries of Northern and Western Europe, it remained below 5%. Poland has one of Europe’s highest percentages of invasive isolates resistant to carbapenems—in 2020 it reached 78.2%. Very high prevalence of resistant isolates was observed for example in Greece—94.6%, Turkiye—93.1% and Italy—80.8%. In comparison, in Germany only 3.5% of isolates were resistant to this group of antibiotics [4,5]. *A. baumannii* is an opportunistic pathogen, especially dangerous for people: hospitalized, with reduced immunity, or having contact with indwelling medical devices. These bacilli mainly cause nosocomial or healthcare-associated infections [6,7]. The most common ones include ventilator-associated pneumonia, urinary tract infections, central line-associated bloodstream infections, surgical site infections, and soft tissue and skin infections [8,9]. The pathogenicity potential of *A. baumannii* strains varies and depends on the pathogenicity factors they produce, which are responsible for the course of the infection. One of the key mechanisms that significantly exacerbates the disease process is the ability to form biofilm [10]. The strains that can produce biofilm pose a greater threat because they are more antibiotic-resistant [11]. Pili and surface-adhesion proteins are essential in *A. baumannii* biofilm initiation and maturation [12].

The growing resistance of these bacteria to antibiotics is the most worrying in the case of infections caused by *A. baumannii*. They are classified as the ESKAPE group, an acronym referring to *Enterococcus faecium*, *Staphylococcus aureus*, *Klebsiella pneumoniae*, *A. baumannii*, *Pseudomonas aeruginosa*, and *Enterobacter* spp. [13]. All these bacteria cause nosocomial infections and are multidrug-resistant (MDR). Clinical isolates of *A. baumannii* are often characterized by resistance to commonly used antibiotics [14,15,16]. They are also frequently resistant to carbapenems used to treat infections caused by MDR strains [17]. In 2017, the World Health Organization (WHO) identified the carbapenem-resistant *A. baumannii* as a pathogen for which novel antimicrobial therapeutic strategies are necessary [18]. Strains that exhibit total resistance to carbapenems often show several resistance mechanisms simultaneously. These mechanisms include reduced permeability of the outer membrane resulting from reduced porin expression, the presence of efflux pumps that remove antibiotics from the cells, and the production of hydrolytic enzymes, such as extended-spectrum β-lactamases (ESBLs) and carbapenemases [19]. β-lactamases produced by *Acinetobacter* belong to classes A, B, C, and D according to Ambler [20]. However, the most common enzymes are class D—CHDL (carbapenem-hydrolysing class D β-lactamases). Acquired oxacillinases can belong to five groups: OXA-23-like, OXA-40-like, OXA-58-like, OXA-143-like, and OXA-235-like. Enzymes of the remaining classes are less common [21].

The study aimed to characterize clinical isolates of *A. baumannii* from three hospitals in various regions of Poland. Their antibiotic susceptibility profile and the presence of genes encoding carbapenemases were determined. Moreover, the occurrence of virulence genes associated with adhesion, biofilm formation, glycoconjugates, fimbriae, siderophore synthesis, host cell death, and toxin production was determined in these isolates.

## 2. Materials and Methods

### 2.1. Bacterial Isolates

A total of 90 clinical isolates of *A. baumannii* were included in this study. The isolates were obtained from patients hospitalized in three hospitals in Poland: in Szczecin (30), Łódź (27), and Wołomin (33), located in the West Pomeranian, Lodz, and Masovian Voivodeships, respectively. The isolates were collected in 2024 as part of routine diagnostic microbiology conducted in these hospitals and were identified by MALDI-TOF technique. Based on the Medical Microbiological Laboratory database, single *A. baumannii* isolates from different patients who showed symptoms of infection were used in the study. The patients were hospitalized mainly in Internal Medicine, Intensive Care, Surgery, Nephrology, Orthopedics, etc. Isolates were obtained from wounds (35), bronchial aspirate (15), blood (11), anal swab (11), urine (8), bronchoalveolar lavage (BAL) (6) and sputum (4). The origin of isolates, considering the Poland region, is shown in Table 1.

### 2.2. Detection of Antibiotic Resistance

Resistance to antibiotics was performed using the disc-diffusion method according to the guidelines of the European Committee on Antimicrobial Susceptibility Testing (EUCAST 2024). The following antibiotics were applied: penicillins: ampicillin with sulbactam (SAM, 10 μg + 10 μg), piperacillin with tazobactam (TZP, 100 μg + 10 μg); cephalosporins: ceftazidime (CAZ, 30 μg), cefepim (FEP, 30 μg); carbapenems: imipenem (IMP, 10 μg), meropenem (MEM, 10 μg); aminoglycosides: amikacin (AK, 30 μg), gentamicin (GM, 10 μg); tobramycin (TOB, 10 μg); fluoroquinolones: ciprofloxacin (CIP, 5 μg), levofloxacin (LEV, 5 μg); other drugs: trimethoprim plus sulfamethoxazole (SXT, 1.25 μg + 23.75 μg). *Pseudomonas aeruginosa* ATCC 27853 was used as a control strain. Results were interpreted according to EUCAST guidelines-15.0 and CLSI (Clinical & Laboratory Standards Institute) guidelines [22,23].

### 2.3. DNA Isolation

DNA from bacterial cells was isolated with the use of the Genomic Micro AX Bacteria+ Gravity (A&A Biotechnology, Gdańsk, Poland) or the NucleoSpin Microbial DNA (Macherey-Nagel GmbH & Co., KG, Düren, Germany) according to the manufacturer’s protocol.

### 2.4. Detection of Resistance and Virulence Genes

Genes conditioning the resistance to carbapenems of class A: *bla*_KPC_, *bla*_IMI_, *bla*_GES_; class B: *bla*_VIM_, *bla*_IMP_, *bla*_GIM_, *bla*_NDM_ as well as class D: *bla*_OXA-48_, *bla*_OXA-23_, *bla*_OXA-40_, *bla*_OXA-58_, ISAba-*bla*_OXA-51_ as well as the gene associated with bacterial adhesion (*espA*, *bap*, *ata*, *chop*), biofilm formation (*ompA*, *pbpG*), siderophore synthesis (*basD*), glycoconjugates (*bfmR*), host cell death (*fhaB*, *abeD*), toxin production (*cpaA*, *lipA*), and stress response (*recA*) were detected by PCR according to the method given by Cerezales et al. and Park et al. [24,25]. PCR products were detected by electrophoresis (70 V, 1.5 h) in 1% (*w*/*v*) agarose gels with Midori Green DNA stain (NIPPON Genetics EUROPE, Düren, Germany) and visualized using UV light (Syngen Imagine, Syngen Biotech, Wrocław, Poland). PCR product size was determined using the marker DraMix (A&A Biotechnology, Gdynia, Poland).

### 2.5. Biofilm Formation Assay

The isolates were grown on Tryptic-Soy Agar (TSA; BBL, Becton Dickinson, Sparks, MD, USA) with 1% glucose at 37 °C for 18 h. Next, bacterial cells were transferred to Tryptic-Soy Broth (TSB) with 1% glucose to prepare a cell suspension containing about 10^8^ CFU/mL. The bacterial cell suspension (200 µL) was inoculated in three replicates into wells of a tissue culture polystyrene 96-well plate (Nunclon, Roskilde, Denmark). The study of biofilm formation by *A. baumannii* was performed according to the method given by Piechota et al. (2018) [26]. Each assay was performed three times and results were averaged. Values of absorbance ≥ 0.12 were regarded as biofilm-positive, <0.2 were considered weak producers, 0.2–0.4 were moderate producers, and >0.4 were considered strong producers.

### 2.6. Statistical Analysis

The chi-square test was used to determine the relationship between the antibiotic resistance of *A. baumannii* and the region of Poland, the hospital ward, and the clinical material from which the isolates originated. This test was also used to check the relationship between the occurrence of resistance and virulence genes, the ability to form biofilm, the region, the hospital ward, and the clinical material from which the isolates originated. The level of *p* ≤ 0.05 was regarded as significant. For the statistical analysis, STATISTICA 13.1 PL software was applied (StatSoft 2016, Cracow, Poland).

## 3. Results

### 3.1. Resistance to Antibiotics

**Figure 1:**
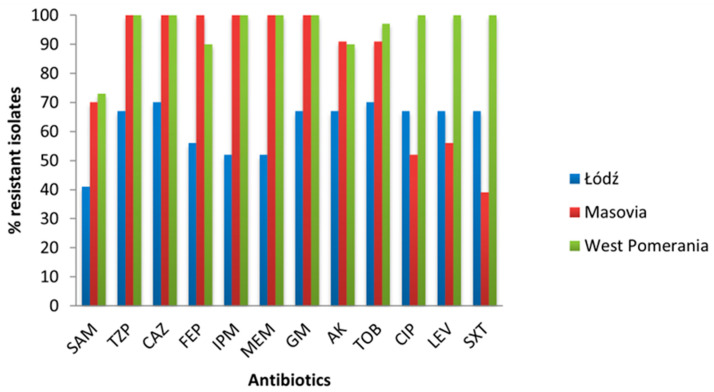
Antimicrobial resistance of *A. baumannii* isolates from different regions in Poland. SAM—ampicillin with sulbactam, TZP—piperacillin with tazobactam, CAZ—ceftazidime, FEP—cefepim, IPM—imipenem, MEM—meropenem, GM—gentamicin, AK—amikacin, TOB—tobramycin, CIP—ciprofloxacin, LEV—levofloxacin, SXT—trimethoprim plus sulfamethoxazole.

More than half of the 90 *A. baumannii* isolates (52.2%) were resistant to all tested antibiotics (Table 2). All isolates from patients hospitalized in West Pomerania and Masovia were MDR because they showed resistance to at least nine antibiotics, including carbapenems (Table 2, Figure 1). Meanwhile, among isolates from Łódź, 25.9% were sensitive to all antibiotics, and 48.1% of isolates from this region were susceptible to meropenem and imipenem (Figure 1).

All *A. baumannii* isolates from bronchial aspirate, anal swab, blood and urine were resistant to meropenem and imipenem as well as to piperacillin with tazobactam, ceftazidime, cefepim, gentamicin, ciprofloxacin, levofloxacin, and trimethoprim plus sulfamethoxazole. The lower percentage of resistant isolates to carbapenems was observed among isolates from BAL (83.3%), wound (71.4%), and sputum (50.0%) (Table 3). Some isolates showed sensitivity to ampicillin with sulbactam. Frequency of sensitivity to ampicillin with sulbactam was the highest in a group of isolates from sputum (all isolates were sensitive) and in isolates from wounds (more than half of the isolates), and this was significantly higher than in the other groups of isolates (*p* = 0.01). Moreover, isolates from wound also frequently showed sensitivity to cefepim (*p* = 0.0014) compared with other isolates.

**Figure 2:**
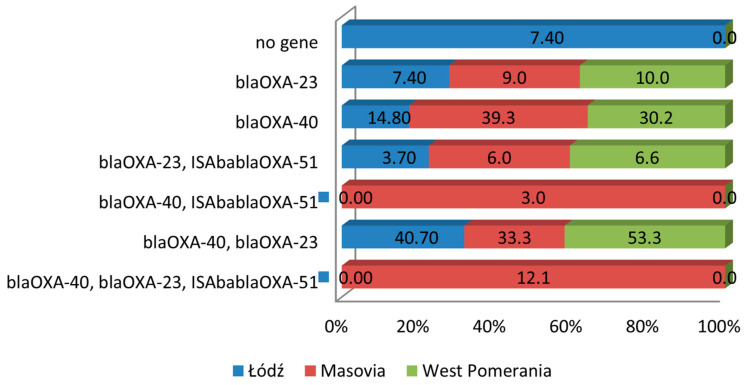
Percentage of isolates with resistance genes in *A. baumannii* isolates from different regions of Poland.

Of the 12 investigated carbapenem resistance genes, only those encoding class D carbapenemases were detected: *bla_OXA-40_*, *bla_OXA-23_*, and ISAba-*bla_OXA-51_*. The *bla_OXA-40_* gene was detected in 83.3% of the investigated isolates. This gene was more frequent in isolates from Masovia (39.3%) and West Pomerania (30.2%) than in isolates from Łódź (14.8%) (Figure 2). Among isolates with *bla_OXA-40_* gene, 33.1% had only this gene. The *bla_OXA-23_* gene was detected in 68.8% of isolates, whereas the ISAba-*bla_OXA-51_* gene was present in 18.9% of the isolates, mainly from Łódź and Masovia, but this relationship was not significant. The ISAba-*bla_OXA-51_* gene was present simultaneously with *bla_OXA-40_*, *bla_OXA-23_*_,_ or with *bla_OXA-40_* and *bla_OXA-23_* (Figure 2).

In 25.9% of isolates from Łódź and 12.1% from West Pomerania, *bla_OXA-40_*, *bla_OXA-23_*, and ISAba-*bla_OXA-51_* were simultaneously present. In two isolates from wounds of patients hospitalized in Łódź, which were sensitive to all investigated antibiotics, carbapenem resistance genes were not detected.

### 3.2. Virulence-Associated Genes in A. baumannii Isolates

Among 13 assessed genes associated with virulence of *A. baumannii*, the *ompA*, *ata*, and *recA*, responsible for biofilm formation, adhesion, and stress response, respectively, occurred in all isolates (Table 4). Other genes associated with adhesion (*bap*—94.4%) and biofilm formation (*pbpG*—90.0%) were also frequently found. Genes responsible for the production of other adhesins *(espA*—4.4%, *chop*—37.7%) were detected less frequently in the study population. The presence of the *basD* gene associated with siderophore synthesis was detected in almost all isolates (97.7%); similarly *lipA* gene encoding extracellular lipase was common in the tested isolates (92.2%), while the *cpaA* gene encoding metalloproteinase was detected only in one isolate from the wound. Other genes assessed, including those responsible for inducing host cell death (*fhaB*—71.1%, *abeD*—93.3%) and production of glycoconjugates (*bfmR*—84.4%), were commonly present in the isolates studied. It was found that in all isolates from anus, the *bap*, *ata*, *ompA*, *pbpG*, *bfmR*, *fhaB*, *abeD*, *basD*, *lipA*, *recA* genes were present, and also *chop* gene was found in 64.2% of isolates (Table 4).

**Figure 3:**
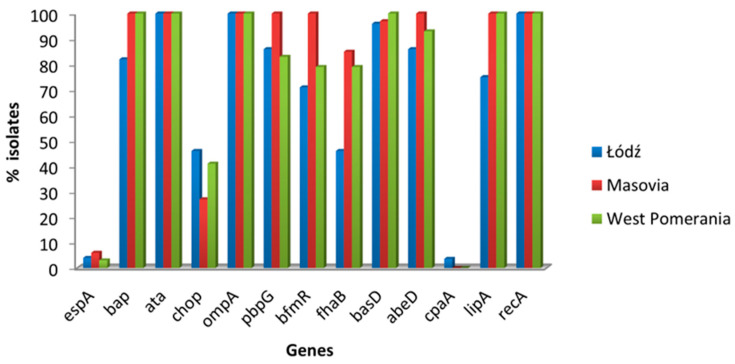
Occurrence of virulence genes in *A. baumannii* isolates from patients from three regions of Poland.

All isolates from patients from Masovia had the *bap*, *ata*, *ompA*, *pbpG*, *bfmR*, *abeD*, *lipA*, and *recA* genes, whereas the *chop* gene responsible for adhesion and invasion of bacteria into host cells was present only in 27.2% of isolates, and it was more common in isolates from other regions. The percentage of the isolates with *bap*, *fhaB*, *basD*, *abeD*, *and lipA* genes from Łódź was lower than in other investigated regions. The frequency of virulence genes in isolates from patients from different regions of Poland is shown in Figure 3.

### 3.3. Biofilm Formation by A. baumannii

**Figure 4:**
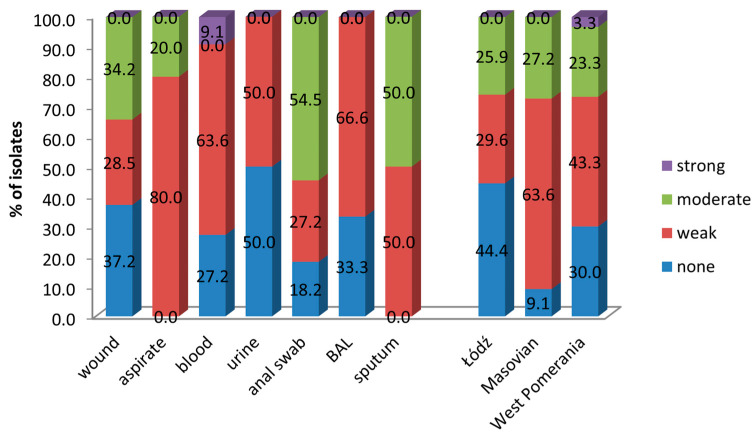
Forming biofilm by *A. baumannii* isolates from different clinical materials and different regions of Poland.

The ability to form biofilm was found in 68.8% of isolates, but in only 1 case, the biofilm was strong. The isolates showed different degrees of adherence to polystyrene microtiter plates (Figure 4). However, no significant differences were observed in the degree of biofilm formation by the isolates from various clinical materials. On the other hand, the isolates from the patients from Masovia formed biofilm more frequently than isolates from other regions (*p* = 0.001).

Statistical analysis showed a significant relationship between the presence of virulence genes *(chop*, *p* = 0.01; *pbpG*, *p* = 0.0001; *bfmR*, *p* = 0.03; *fhaB*, *p* = 0.03; *abe*, *p* = 0.0003) and the ability to form biofilm. There was also a strong relationship between the presence of virulence genes (at least 9) and the presence of the *bla_OXA-23_* gene (*p* = 0.009).

## 4. Discussion

Currently, the great challenge is to stop the spread of multidrug resistance in bacteria. This problem concerns the whole world and generates high treatment costs. According to the literature, the resistance to antibiotics is increasing among the microorganisms, including *A. baumannii* [27]. The scale of resistance to carbapenems in these bacteria depends on the region from which they are isolated. The greatest problem concerns Asia and the Americas and areas surrounding the Mediterranean [3]. Between 2013 and 2017, resistance to carbapenems in *A. baumannii* in Europe concerned 35.6% of isolated strains. However, there is a large diversity in individual regions of Europe. In Southern and Eastern Europe, the percentage of resistant isolates can be as high as 75.5% and 71.5%, while in the Northern and Western regions these values are 2.8% and 6.3%, respectively [28]. The percentages of carbapenem-resistant *Acinetobacter* spp. varied widely within Europe in 2020, from below 1.0% in Ireland, the Netherlands and Norway to up to 50.0% or above in 21 countries/areas, mostly in southern and eastern Europe [5].

We have tested the resistance to antibiotics in *A. baumannii* isolated in 2024 from hospitalized patients from three hospitals in Poland, located in the West Pomerania, Łódź and Masovian Voivodeship. Most isolates came from wounds and bronchial aspirate. Of the 90 *A. baumannii* isolates, 85.5% were MDR, because they showed resistance to at least three classes of antimicrobials. However, there was a large variation in the occurrence of resistance between isolates from different regions of Poland. In West Pomerania and Masovia all isolates were MDR, while in Łódź, 51.8% of isolates showed MDR. Furthermore, it should be added that all isolates from patients from hospitals in West Pomerania and Masovia were resistant to at least nine antibiotics. Carbapenem resistance of *A. baumannii* isolates from patients hospitalized in seven hospitals in southern Poland in 2019–2021 was 69% [29]. Our results demonstrate increasing resistance to this group of antibiotics in Poland. As a result, new therapeutic options are needed to treat severe infections caused by *Acinetobacter* spp. Our studies show that some isolates were sensitive to ampicillin with sulbactam. The importance of sulbactam, which inhibits the degradation of β-lactams by β-lactamases, is more and more highlighted in the literature [30,31,32]. Administration of high-dose ampicillin-sulbactam in combination with another agent can significantly reduce mortality in the intensive care unit patients [30,32,33]. Of the twelve carbapenemase genes we tested, only three were detected. The predominant ones were *bla*_OXA-40_ (83.3%) and *bla*_OXA-23_ (68.8%). Moreover, 42.2% of the isolates had both of these genes at the same time. The *bla*_OXA-40_-like β-lactamases exhibit weak hydrolytic properties against cephalosporins and carbapenems, such as imipenem and ceftazidime, while *bla*_OXA-23_-like β-lactamases are associated with resistance to ticarcillin, meropenem, amoxicillin, and imipenem [34]. These genes can be part of the chromosome or located on plasmids, which means they can spread easily in the environment [35]. Our results confirm the dominance of these genes among *A. baumannii* isolates in Poland [29,36]. However, the most common CHDL enzymes in *A. baumannii* worldwide are *bla*_OXA-23_-like β-lactamases. Isolates producing these enzymes have been described in many countries [37,38,39]. A total of 18.8% of isolates tested by us had the ISAba-*bla*_OXA-51_ gene. It always existed together with other carbapenem resistance genes. The CHDL enzyme from the OXA-51-like group naturally exists in all *A. baumannii* isolates. However, it gives low activity against carbapenems. Only the presence of the strong ISAba1 promoter causes its overexpression and reduces the sensitivity of isolates to this group of antibiotics [40]. Due to the fact that isolates we studied were MDR, we did not observe any correlation between phenotypic antibiotic resistance and the presence of specific carbapenem resistance genes. However, isolates with the *bla*_OXA-23_ gene were more virulent, harboring at least nine virulence genes in the genome. We did not detect the remaining resistance genes we were looking for, but they had been previously described in *A. baumannii* [41,42,43,44]. We also checked whether there was a relationship between the ability to produce biofilm and antibiotic resistance. Such correlations in *A. baumannii* had been previously observed [45,46]. A total of 68.8% of the isolates we tested produced biofilm, of which only one formed a strong biofilm. Similar results were obtained by Asaad et al., of the strains they tested [46]. On the other hand, the studies conducted by Kasperski et al., in which 100% of isolates produced biofilm, of which as many as 72.0% were strong, are in contrast [29]. We have not identified any correlation between the ability to produce biofilm and the resistance to antibiotics. What is worth highlighting is that Qi et al. indicated a negative correlation between biofilm formation and antibiotic resistance. According to their studies, non-MDR isolates were stronger biofilm producers than MDR or XDR ones [47]. In the studies conducted by Rodrigez-Bano et al. biofilm-forming isolates were less frequently resistant to imipenem and ciprofloxacin [48].

We investigated the presence of virulence genes important in the initiation and development of the infection. To our knowledge, this is a first report analyzing the occurrence of such a large number of resistance and virulence genes in *A. baumannii* isolated from clinical material in Poland. In all tested isolates present was the stress response-associated *recA* as well as the *ompA* i *ata* genes encoding proteins that play a role in adhesion and biofilm formation. OmpA directly adheres and invades the host cells, which may lead to cell apoptosis through the destruction of nucleus and mitochondria [49,50]. Higher expression of the *ompA* gene was demonstrated in *A. baumannii* isolates from patients with pneumonia and bacteremia, which may suggest an association of OmpA with a severe course of infection and higher mortality [51]. The *ata* gene also plays an important role in biofilm production. One of the domains of *Acinetobacter* trimeric autotransporter Ata is an adhesin that binds proteins of the host extracellular matrix, and inactivation of the *ata* gene may reduce biofilm formation on a plastic surface [52]. This protein also mediates invasion, host cell modulation and apoptosis in endothelial and epithelial cells. Phylogenetic profiling in 3052 *Acinetobacter* spp. genomes available in the NCBI RefSeq revealed that *ata* is present in 78% of all sequenced *A. baumannii* [53]. In the process of formation and maturation of biofilms, an important role is also played by the Bap protein encoded by *bap* gene, which was present in 94.4% of isolates we examined. Mutations in the encoding gene of this protein impair the adhesion abilities of *A. baumannii* [54]. Other studies confirm the common occurrence of *ata* and *bap* genes of *Acinetobacter* [29,55,56]. In the studies of Depka at al., which also concerned *A. baumannii* from patients from Poland, 100% of isolates had the *bap* gene [57]. The presence of *bap* gene in isolates may be related to the site of infection caused by *A. baumannii*. In our studies, this gene was not detected in isolates from wounds and sputum, and these isolates came from only from patients from the Łódź Voivodeship. In spite of the lack of this gene, they were forming biofilm. For survival of *A. baumannii* under stressful conditions, the BfmRS two-component regulatory system is indispensable [58]. This system serves many functions in the bacterial cell, including being involved in the adhesion of bacteria to abiotic surfaces and biofilm formation. A more important role in this process is assigned to BfmR [59]. Moreover, BfmR and BfmS may be associated with the resistance to different antibiotics, including carbapenems. Marr et al. demonstrated that loss of BfmR caused increase in the sensitivity to meropenem in the XDR *A. baumannii* strain tested by them [60]. According to Geisinger et al., this may be related to increased production of β-lactams and cell envelope synthesis regulated by bfmR [61]. In our studies, the *bfmR* gene was detected in 84.4% of isolates, including all those from Mazovia. We have not observed a correlation between the presence of the *bfmR* gene and antibiotic resistance, but statistical analysis showed a significant relationship between the presence of this gene and the ability to form biofilm (*p* = 0.03). In other works, this gene occurred in all of the tested isolates [62,63]. In the bacteria adhesion to cells, the FhaB/FhaC two-partner secretion system is also involved [64]. The *fhaB* gene was identified in 71.1% of the isolates tested by us, and its presence was correlated in forming biofilm (*p* = 0.03). The studies made by Perez et al. confirmed that inactivation of the AbFhaB/FhaC system decreases bacterial attachment to epithelial cells. Simultaneously, in the strains analyzed by them no relationship between the capacity to form biofilms and eukaryotic cell adherence was demonstrated [64]. On the other hand, the results of Astaneh et al. demonstrated association of FhaB1with bacterial adhesion and biofilm formation [65]. We have also examined the occurrence of the *pbpG* gene encoding the penicillin-binding protein, which plays an important role in the integrity of the *A. baumannii* cell wall and the survival of the bacterium within the host. This gene occurred in 90.0% of the tested strains and was associated with biofilm formation (*p* = 0.0001). Similar results were obtained in the work of Mozafari et al. [63]. Loss of PBP 7/8 increases susceptibility to complement and lysozyme activity and increases bacterial membrane permeability to antimicrobial substances [66]. In most of the tested isolates (97.7%) the *basD* gene was present. It is an important element of the iron acquisition system by *A. baumannii* and plays a key role in the synthesis of the siderophore acinetobactin [67]. Other studies also confirm its frequent occurrence in *Acinetobacter* spp. [57,63]. In contrast to these results is the work of Porbaran et al., in which only 12.5% of the tested isolates had the *basD* gene [68]. In our study, 33.3% of isolates from Masovia, 30.0% from West Pomerania and 18.5% from the Łódź had 11 virulence genes each. Nevertheless, isolates from patients from Masovia had more virulence genes than isolates from the other two regions. All had the following set of genes: *bap*, *ata*, *ompA*, *pbpG*, *bfmR*, *abeD*, *lipA* and *recA*. On the other hand, the *espA* and *cpaA* genes were present in single isolates. Only 0.6% of the strains studied by Park et al. had the *cpaA* gene, but the *espA* gene was detected more often than in our study [24]. The proteins encoded by these genes play an important role in the virulence of *Acinetobacter* spp. The CpaA protein is a metalloproteinase leading to reduced factors V and XII, while the EspA protein is an exopolysacharide, occurring on cell surfaces and participating in biofilm and capsule formation [63,69,70]. On the other hand, the gene encoding the ChoP protein, which facilitates bacterial entry into epithelial cells through contact with the platelet-activating factor receptor (PAFR) on the host cell surfaces, was present in our study in 37.7% of isolates, all of which were from anal swabs. In the study by Park et al., this gene was present in only 7.1% of strains isolated from blood [24]. We have tested the occurrence of the *lipA* gene encoding the enzyme necessary for the decomposition of lipids obtained exogenously [71]. It was present in 92.2% of the isolates investigated by us, including all these from West Pomerania and Masovia. The *abeD* gene occurred with similar frequency (93.3%). In the study of Suh et al., over 99% of isolates had this gene, which takes part in killing host cell [72]. In our study, this gene was present in all isolates from Masovia. These results underline higher adaptive abilities of the bacteria isolated from patients of this voivodeship and their greater virulence.

## 5. Conclusions

In this study, we evaluated the antibiotic resistance profile, biofilm production, and the frequency of genes encoding carbapenemases and virulence factors in *A. baumannii* isolates from patients of three hospitals in various regions of Poland. We found that more than half of isolates were MDR because they showed resistance to penicillins with β-lactamase inhibitors, cephalosporins, carbapenems, aminoglycosides, fluoroquinolones, and trimethoprim plus sulfamethoxazole. Moreover, we found that the *bla_OXA-23_* (68.8%), *bla_OXA-40_* (83.3%), and ISAba-*bla*_OXA-51_ (18.8%) genes encoding carbapenemases were present in this population. In investigated population of *A. baumannii*, we found isolates with genes responsible for biofilm formation, adhesion, stress response, production of siderophore, toxins, glycoconjugates and inducing host cell death. It turned out that isolates from three different regions of Poland showed different sensitivity to antibiotics and different virulence potential. The isolates from patients from Masovia had more virulence genes than isolates from the other two regions; moreover, all isolates from patients hospitalized in Masovia and West Pomerania were MDR including resistance to carbapenems. Our studies have shown that in some regions of Poland, all *A. baumannii* isolates from hospitalized patients are MDR, including carbapenem-resistant, and have a high virulence potential. These isolates pose a serious threat to the health of patients, and the treatment of infections caused by them requires new antimicrobial therapeutic strategies.

## Figures and Tables

**Table 1:** The origin of *A. baumannii* isolates used in this study.

Source of Isolates	Number of Isolates from the Voivodeship:			
	Łódź	West Pomeranian	Masovian	Total
Wound	18	9	8	35
Bronchial aspirate	0	0	15	15
Blood	4	4	3	11
Anal swab	0	6	5	11
Urine	2	4	2	8
BAL	1	5	0	6
Sputum	2	2	0	4
**Total**	**27**	**30**	**33**	**90**

BAL—bronchoalveolar lavage.

**Table 2:** Resistance patterns of *A. baumannii* isolates from different regions of Poland.

Resistance Phenotypes (No. of Antibiotics)	Number (%) of Isolates from:			
	Masovia	West Pomerania	Łódź	Total
**(12)**				
SAM, TZP, CAZ, FEP, IPM, MEM, GM, AK, TOB, CIP, LEV, SXT	21 (63.6)	18 (60.0)	8 (29.6)	47 (52.2)
**(11)**				
TZP, CAZ, FEP, IPM, MEM, GM, TOB, CIP, LEV, SXT	9 (27.3)	6 (20.0)	3 (11.1)	18 (20.0)
SAM, TZP, CAZ, FEP, IPM, MEM, GM, TOB, CIP, LEV, SXT	0 (0.0)	2 (6.7)	0 (0.0)	2 (2.2)
SAM, TZP, CAZ, IPM, MEM, GM, AK, TOB, CIP, LEV, SXT	0 (0.0)	1 (3.3)	1 (3.7)	2 (2.2)
**(10)**				
SAM, TZP, CAZ, FEP, IPM, MEM, GM, CIP, LEV, SXT	2 (6.1)	1 (3.3)	0 (0.0)	3 (3.3)
SAM, TZP, CAZ, FEP, GM, AK, TOB, CIP, LEV, SXT	0 (0.0)	0 (0.0)	1 (3.7)	1 (1.1)
TZP, CAZ, IPM, MEM, GM, AK, TOB, CIP, LEV, SXT	0 (0.0)	2 (6.7)	2 (7.4)	4 (4.4)
**(9)**				
TZP, CAZ, FEP, GM, AK, TOB, CIP, LEV, SXT	0 (0.0)	0 (0.0)	2 (7.4)	2 (2.2)
TZP, CAZ, FEP, IPM, MEM, GM, CIP, LEV, SXT	1 (3.0)	0 (0.0)	0 (0.0)	1 (1.1)
**(6)**				
CAZ, GM, AK, CIP, LEV, SXT	0 (0.0)	0 (0.0)	1 (3.7)	1 (1.1)
**(5)**				
SAM, TZP, CAZ, FEP, TOB	0 (0.0)	0 (0.0)	1 (3.7)	1 (1.1)
**(1)**				
TOB	0 (0.0)	0 (0.0)	1 (3.7)	1 (1.1)

SAM—ampicillin with sulbactam, TZP—piperacillin with tazobactam, CAZ—ceftazidime, FEP—cefepim, IPM—imipenem, MEM—meropenem, GM—gentamicin, AK—amikacin, TOB—tobramycin, CIP—ciprofloxacin, LEV—levofloxacin, SXT—trimethoprim plus sulfamethoxazole.

**Table 4:** The prevalence of virulence-associated genes in *A. baumannii* isolates from different clinical materials.

Source	Virulence Genes, No. (%)												
	*espA*	*bap*	*ata*	*chop*	*ompA*	*pbpG*	*bfmR*	*fhaB*	*abeD*	*basD*	*cpaA*	**lipA**	**recA**
**Wound** *n* = 35	1 (2.8)	32 (91.4)	35 (100)	15 (42.8)	35 (100)	31 (88.5)	27 (77.1)	21 (60.0)	30 (85.7)	34(97.1)	1 (4.1)	32 (91.4)	35 (100)
**Bronchial aspirate** *n* = 15	1 (6.6)	15 (100)	15 (100)	3 (20.0)	15 (100)	15 (100)	15 (100)	12 (80.0)	15 (100)	14(93.3)	0 (0.0)	15 (100)	15 (100)
**Anal swab** *n* = 11	0 (0.0)	11 (100)	11 (100)	7 (63.6)	11 (100)	11 (100)	11 (100)	11 (100)	11 (100)	11 (100)	0 (0.0)	11 (100)	11 (100)
**Blood** *n* = 11	0 (0.0)	11 (100)	11 (100)	4 (36.3)	11 (100)	9 (81.8)	10 (90.9)	8 (72.7)	11 (100)	11 (100)	0 (0.0)	10 (90.9)	11 (100)
**Urine** *n* = 8	1 (12.5)	8 (100)	8 (100)	1 (12.5)	8 (100)	7 (87.5)	6 (75.0)	6 (75.0)	7 (87.5)	8 (100)	0 (0.0)	8 (100)	8 (100)
**BAL** *n* = 6	1 (16.6)	6 (100)	6 (100)	2 (33.3)	6 (100)	4 (66.6)	3 (50.0)	4 (66.6)	6 (100)	6 (100)	0 (0.0)	6 (100)	6 (100)
**Sputum** *n* = 4	0 (0.0)	2 (50.0)	4 (100)	2 (50.0)	4 (100)	4 (100)	4 (100)	2 (50.0)	4 (100)	4 (100)	0 (0.0)	2 (50.0)	4 (100)
**Total**	**4 (4.4)**	**85 (94.4)**	**90 (100)**	**34 (37.7)**	**90 (100)**	**81 (90.0)**	**76 (84.4)**	**64 (71.1)**	**84 (93.3)**	**88 (97.7)**	**1 (1.1)**	**83 (92.2)**	**90 (100)**

**Table 3:** The prevalence of antimicrobial resistance in *A. baumannii* isolates from different clinical materials.

Source	No. (%) of Resistant Isolates to Antimicrobials											
	SAM	TZP	CAZ	FEP	IPM	MEM	GM	AK	TOB	CIP	LEV	SXT
**Wound** *n* = 35	17 (48.6)	28 (80.0)	29 (82.9)	23 (65.7)	25 (71.4)	25 (71.4)	29 (82.8)	26 (74.3)	27 (77.1)	29 (82.8)	29 (82.8)	29 (82.8)
**Bronchial aspirate** *n* = 15	11 (73.3)	15 (100)	15 (100)	15 (100)	15 (100)	15 (100)	15 (100)	14 (93.3)	14 (93.3)	15 (100)	15 (100)	15 (100)
**Anal swab** *n* = 11	9 (81.8)	11 (100)	11 (100)	11 (100)	11 (100)	11 (100)	11 (100)	10 (90.9)	11 (100)	11 (100)	11 (100)	11 (100)
**Blood** *n* = 11	9 (81.8)	11 (100)	11 (100)	11 (100)	11 (100)	11 (100)	11 (100)	10 (90.9)	10 (90.9)	11 (100)	11 (100)	11 (100)
**Urine** *n* = 8	5 (62.5)	8 (100)	8(100)	8(100)	8 (100)	8 (100)	8(100)	8(100)	8(100)	8(100)	8(100)	8(100)
**BAL** *n* = 6	5 (83.3)	6 (100)	6(100)	5 (83.3)	5 (83.3)	5(83.3)	5 (83.3)	5 (83.3)	6(100)	5 (83.3)	5 (83.3)	5 (83.3)
**Sputum** *n* = 4	0(0.0)	2 (50.0)	2 (50.0)	2 (50.0)	2 (50.0)	2(50.0)	2 (50.0)	2 (50.0)	2 (50.0)	2 (50.0)	2 (50.0)	2 (50.0)

SAM—ampicillin with sulbactam, TZP—piperacillin with tazobactam, CAZ—ceftazidime, FEP—cefepim, IPM—imipenem, MEM—meropenem, GM—gentamicin, AK—amikacin, TOB—tobramycin, CIP—ciprofloxacin, LEV—levofloxacin, SXT—trimethoprim plus sulfamethoxazole.

## Data Availability

The original contributions presented in this study are included in the article. Further inquiries can be directed to the first author.
